# A Comprehensive Review of Vision-Based Sensor Systems for Human Gait Analysis

**DOI:** 10.3390/s25020498

**Published:** 2025-01-16

**Authors:** Xiaofeng Han, Diego Guffanti, Alberto Brunete

**Affiliations:** 1 Centre for Automation and Robotics (CAR UPM-CSIC), Escuela Técnica Superior de Ingeniería y Diseño Industrial (ETSIDI), Universidad Politécnica de Madrid, Ronda de Valencia 3, 28012 Madrid, Spain; alberto.brunete@upm.es; 2 Universidad UTE, Av. Mariscal Sucre, Quito 170129, Ecuador; diego.guffanti@ute.edu.ec

**Keywords:** human gait analysis, visual sensors, machine learning algorithms, gait parameters, 3D camera, mobile robot

## Abstract

Analysis of the human gait represents a fundamental area of investigation within the broader domains of biomechanics, clinical research, and numerous other interdisciplinary fields. The progression of visual sensor technology and machine learning algorithms has enabled substantial developments in the creation of human gait analysis systems. This paper presents a comprehensive review of the advancements and recent findings in the field of vision-based human gait analysis systems over the past five years, with a special emphasis on the role of vision sensors, machine learning algorithms, and technological innovations. The relevant papers were subjected to analysis using the PRISMA method, and 72 articles that met the criteria for this research project were identified. A detailing of the most commonly used visual sensor systems, machine learning algorithms, human gait analysis parameters, optimal camera placement, and gait parameter extraction methods is presented in the analysis. The findings of this research indicate that non-invasive depth cameras are gaining increasing popularity within this field. Furthermore, depth learning algorithms, such as convolutional neural networks (CNNs) and long short-term memory (LSTM) networks, are being employed with increasing frequency. This review seeks to establish the foundations for future innovations that will facilitate the development of more effective, versatile, and user-friendly gait analysis tools, with the potential to significantly enhance human mobility, health, and overall quality of life. This work was supported by [GOBIERNO DE ESPANA/PID2023-150967OB-I00].

## 1. Introduction

**Figure 1:**
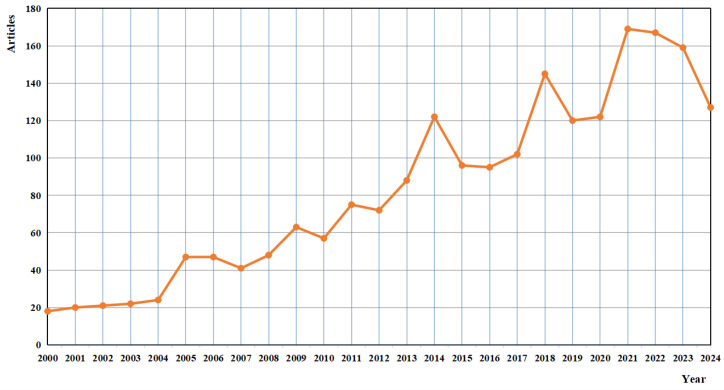
The number of articles published annually on the subject of human gait analysis between the years 2000 and 2024, as collated from the Scopus database.

Analysis of the human gait has been a long-standing area of interest within the fields of biomechanics, clinical research, and a range of interdisciplinary studies. Especially in the past five years, a great number of articles have been published on human gait analysis, as shown in Figure 1. The study of how humans walk, run, and move serves not only as a fundamental aspect of understanding human locomotion, but it also holds immense practical implications in healthcare, rehabilitation, sports science, security, and beyond [1]. Due to the rapid development of science and technology, especially the advances in the field of visual sensors, the field of human gait analysis has been greatly transformed, ushering in a new era of accuracy, objectivity, and applicability.

The human gait is a highly complex phenomenon that is influenced by a variety of factors, including biomechanical structures, neural processes, environmental conditions, and individual features [2]. In the past, analysis of the human gait mainly relied on human observation, subjective assessment by experts, and some simple instrument. However, these methods have many limitations. providing only limited insights and poor repeatability. With the rapid development of visual sensors such as cameras, depth sensors, and infrared imaging devices, a non-invasive, high-resolution approach to capturing and analyzing human gait movements has been provided, revolutionizing the field [3].

Compared to traditional gait analysis methods, visual sensor-based gait analysis has several distinct advantages. Firstly, visual sensors can collect more detailed spatial and temporal data and human gait information, allowing for a more comprehensive characterization of the human gait. For example, high-speed cameras like the Photron FASTCAM SA-Z can capture human motion at hundreds or even thousands of frames per second [4]. Depth sensors, on the other hand, help to extract three-dimensional (3D) information, enabling precise measurements of joint angles, limb trajectories, and body postures [5].

Deep learning algorithms play an important role in the field of vision-based human gait analysis, especially in processing gait data and visual sensor data [6]. These algorithms, especially models based on convolutional neural networks (CNNs) and recurrent neural networks (RNNs), are able to automatically learn hierarchical features from raw visual data, reducing the need for manual feature extraction. Deep learning algorithms are able to process large amounts of complex data, which improves the accuracy of gait recognition, allowing the system to recognize small changes in gait that may be difficult to detect in traditional methods.

In addition, vision sensor-based gait analysis provides an intelligent non-invasive solution for continuous monitoring of human locomotion, which is highly valuable in clinical environments and real-world applications. Unlike wearable sensors or instrumented walkways, visual sensors can capture gait data from subjects in real-time onsite, allowing for more ecologically valid assessments. It is not affected by the laboratory environment and does not require subjects to alter their natural gait patterns [7]. This highlights the growing importance of reviewing the latest technologies within gait analysis, with an accent on how the upcoming technologies (mainly vision systems) are defining this field. The evolution of gait analysis accommodates and follows best practices as new trends, innovations, and opportunities continue to impact in this direction. These innovations not only improve the efficiency and adaptability of gait identification, but also provide new directions for research and applied practice in a variety of fields. Table 1 provides a limited sense of the state of reviews on human gait analysis to offer a holistic orientation, along with the limitations in previous reviews.

The motivation for this review is based on the fact that visual sensor technology and machine learning algorithms have come a long way in the last five years. And that the integration of these technologies has significantly improved the performance of gait analysis systems, making them more accurate and efficient. The aim of this review is to bring together the latest findings from research and the literature, recent scientific ideas, knowledge related to visual sensor-based gait analysis, as well as some of the current challenges and future directions. The main contributions of this paper are as follows.

1. Summary of the latest advances in vision sensor-based gait analysis systems, covering technological innovations and advances in hardware and software from 2019 to 2024.
2. Key algorithms for human gait analysis, feature extraction, and classification are discussed, including advanced machine learning models such as CNNs (convolutional neural networks), LSTMs (long short-term memory Networks), and hybrid models.
3. Different types of vision sensors such as 2D cameras, 3D cameras, and markerless motion capture systems are evaluated and their advantages, limitations, and suitability for various applications in human gait analysis are, respectively, discussed.
4. The important applications of gait analysis in various fields such as healthcare, sports science, and safety through practical examples and case studies are explored.
5. Some of the current challenges and shortcomings are identified, and possible future directions are consolidated and some recommendations are made.

The remainder of this paper is structured as follows: Section 2 describes the survey methodology, including search strategy, study selection, and the inclusion and exclusion criteria. It describes the searched databases and explains how relevant articles were identified, screened, and selected for review. The review results are presented in Section 3, where an overview of the types of vision sensors employed for human gait analysis is given, including RGB cameras, depth sensors, and marker-based and markerless motion capture systems. It reviews the detailed characteristics and performances of these capabilities, as well as algorithms for gait analysis, including CNNs, LSTMs, SVMs, etc. It also offers a detailed examination of gait parameters in spatial, temporal, and kinematic domains.

The last section (Section 4) discusses the findings of this critical review and the implications that technological developments in vision-based gait analysis have on fields such as healthcare, rehabilitation, sports science, and security. The Practical Applications (with Case Study and Example) section reflects on advances in diagnostic accuracy and treatment effectiveness, and it describes the limitations of this review, focusing on possible biases and the nature of included articles, commenting specifically on their implications for generalizability and applicability. The final section presents the current challenges and future work in vision-based gait analysis, responding to the research directions: robust algorithms development; integration of multimodal sensor data; and standardization of data collection and analysis approaches as key areas for future investigation that underscore the needs of ongoing creativity and collaboration among researchers, clinicians, and industry partners.

## 2. Survey Methodology

Four more commonly used academic databases were selected: IEEE, PubMed, Web of Science, and Scopus (see Table 2 for details). As shown in Figure 1, the number of papers related to the topic has surged in the last few years. In order to address the latest technologies, the research papers published from 2019 to March 2024 (the last five years) on vision-based sensor systems for human gait analysis were collected.

The next step was to formulate an article search strategy. After comparing and analyzing different combinations of search terms for retrieval, the terms “human gait”, “camera”, “video”, “walk”, “run”, and “jog” were chosen as the keywords. Then, according to the search rules of different databases and Boolean algorithms, these keywords were combined. The specific queries are shown in Table 2.

After making the preparations, the retrieval process began. To ensure a systematic and unbiased selection of relevant studies, we adopted the Preferred Reporting Items for Systematic Reviews and Meta-Analyses (PRISMA) 2020 methodology for the identification [9], screening, and inclusion of studies. This approach allows us to rigorously screen and assess the literature to ensure that only the most relevant and high quality studies were included.

**Figure 2:**
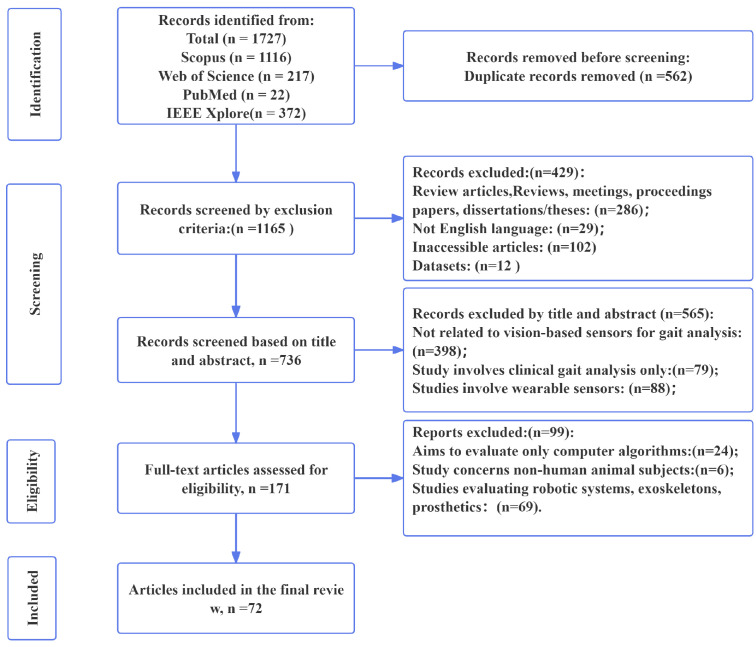
The systematic selection procedure of the studied articles based on PRISMA search strategies. At first, a list of 1727 articles was obtained through a search string from the IEEE Xplore, PubMed, Web of Science, Scopus databases. Finally, 72 articles were selected through the PRISMA steps.

The initial search yielded a total of 1727 articles from these four databases that matched the relevant topics. These articles were collated and downloaded into EndNote for further screening. Firstly, 1165 articles remained after removing duplicates. Then, 171 articles remained after screening based on the inclusion and exclusion criteria in Table 3. Finally, the articles were screened based on the main content of their titles and abstracts, resulting in a total of 72 articles that were included in this review. A statistical analysis of these articles was performed using the Bibliometrix tool of RStudio. The detail of the survey methodology is shown in Figure 2.

A selection of articles has been collated and presented in Table 4 for the reader’s convenience. The table provides a summary of the principal content and characteristics of the selected articles, offering an overview of the various visual sensor systems. In the process of selecting representative articles from the 72 articles initially identified, the following principles were employed to inform the selection: Firstly, the article must utilize a vision-based system for the analysis of human gait, which represents the primary criterion for selection. Secondly, preference is given to articles that utilize a vision-based system in an innovative manner, employ a representative methodology, or provide comprehensive experimental results. This may include, for instance, the introduction of novel algorithms, the evaluation of performance indicators for the vision system, and the level of detail in the analysis of results.

The aforementioned selection method guarantees that the articles included in the table not only encompass the prevailing visual sensor systems, but also exemplify the most recent advancements and applications of these systems in the domain of gait analysis. This approach allows for a clear understanding of the current status of the application of various visual sensor systems in gait analysis and their respective advantages, which provides an important reference point for subsequent research.

## 3. Results

This section presents the findings of a systematic review of visual sensor-based systems for analysis of the human gait. A series of key findings were obtained through comprehensive analyses and summaries of the final selected papers. These include an examination of the types of visual sensors employed, an investigation of the algorithms most commonly utilized, an analysis of the human gait parameters, an evaluation of the optimal camera placement, etc. The most recent advances and current state of development in the field of human gait analysis are presented.

**Figure 3:**
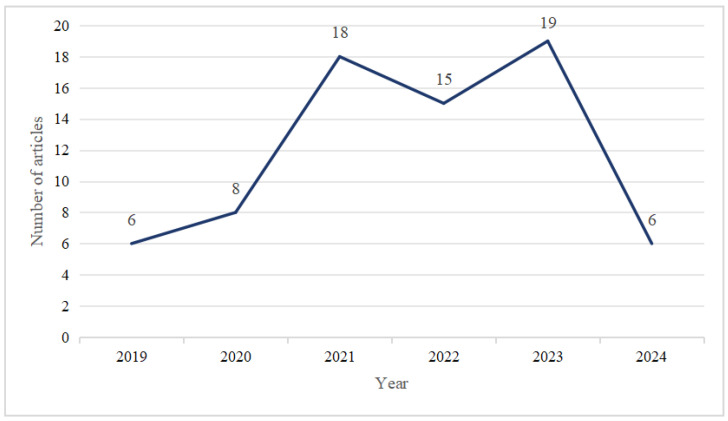
Trends in the number of relevant articles on human gait analysis (2019–2024).

The statistical analysis of the selected articles using the Bibliometrix tool revealed the number of relevant articles published between 2019 and 2024, as shown in Figure 3. Among these 72 articles, the keyword “gait analysis” was the most frequently occurring, followed by “deep learning”.

**Figure 4:**
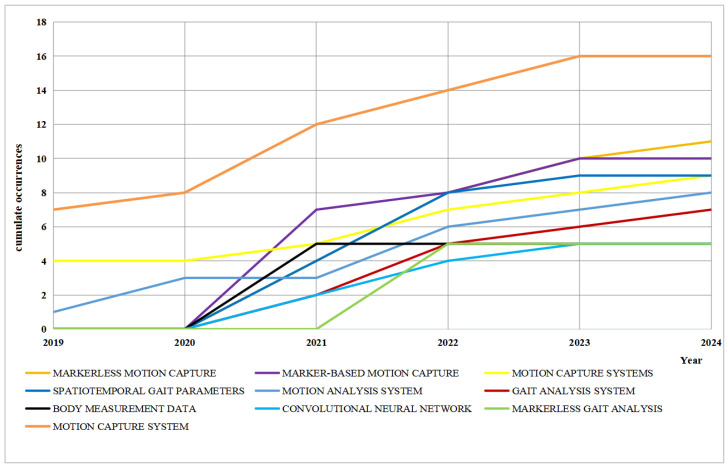
The most frequent phrases in these 72 articles.

As evidenced by Figure 4, recent years have witnessed a notable surge in the cumulative usage frequency of ’markerless motion capture’ technology in human gait analysis systems based on visual sensors. This trend suggests that this technology is gaining traction and becoming increasingly mainstream. Although the use of ‘marked motion capture’ has also increased, the growth rate is relatively slow, indicating a gradual trend of being replaced by markerless technology. Furthermore, the use of motion capture systems and motion analysis systems has maintained a steady increase, indicating that they still play an important role in gait analysis. Additionally, the application of convolutional neural networks (CNNs) is also showing a rapid upward trend, reflecting the growing importance of artificial intelligence in gait analysis.

### 3.1. Types of Vision Sensors

In the field of human gait analysis, visual sensor systems are classified into distinct categories based on their operational principle and modality. Such systems include two-dimensional cameras (RGB), three-dimensional cameras (depth sensors such as Kinect), marker-based motion capture systems, and markerless motion capture systems. Of the 72 articles, 13 are primarily concerned with 2D cameras, 21 with 3D cameras, 12 with marker-based motion capture systems, and 15 with markerless motion capture systems. Each of these data systems demonstrates distinctive advantages and limitations in the capture of gait parameters. This section provides a comprehensive description of these visual sensor systems [31].

#### 3.1.1. 2D Camera

A review of the literature reveals that 2D cameras have already been employed in a number of applications related to human gait analysis. These include clinical diagnosis and rehabilitation, sports biomechanics, and fall risk assessment. Such studies have appeared in recent years, demonstrating the efficacy and innovation of 2D camera systems in gait analysis.

In their 2021 study, Sikandar et al. [32] investigated the potential of two-dimensional image sequences captured by a single camera (no specific camera model was mentioned) to objectively classify human walking speed. Their study introduced five ratio-based body measures extracted from two-dimensional images and employed a deep learning-based bidirectional long short-term memory (LSTM) model to categorize walking speeds into three groups: slow, normal, and fast. The results demonstrate an average classification accuracy of 88.08% and 79.18% in indoor and outdoor environments, respectively. It is noteworthy that the proposed method obviates the necessity of wearing body markers and addresses the issues associated with varying distances between the camera and the participant.

In the field of motion analysis, two-dimensional (2D) video cameras have been employed for the purposes of analyzing running posture and estimating relevant parameters. For example, Scicluna, Seychell, and Spiteri (2023) [33] introduced a new video dataset (Halpe-FullBody) comprising 26 labeled body keypoints. The extant dataset (MPII, COCO-WholeBody) is predominantly image-based and comprises a maximum of 17 key points, which is insufficient for accurately analyzing running posture as it does not include important joints such as the heels and toes. This study emphasizes the significance of utilizing two-dimensional (2D) cameras for the estimation of running posture.

A recent study by Wade et al. (2023) investigated the accuracy of two-dimensional markerless motion capture (1920 × 1080 pixels, JAI sp5000c, JAI Ltd., Copenhagen, Denmark) for measuring joint angles during ground walking. The results showed that, in the sagittal plane, the difference in the angles of the closed side hip and knee joints was approximately twice that of the visible side. In contrast, compared with marker-based motion capture, their unmarked knee joint angle difference in the sagittal plane was 1.5 ± $4.1^{\circ }$ (flexion/extension), and the unmarked knee joint angle difference in the frontal plane was 1.6 ± $4.2^{\circ }$ (adduction/abduction), which was within an acceptable error range. Although the limits of agreement for the frontal plane accounted for 35–46% of the total range of motion for the hip and knee joints, the Bland–Altman bias and limits of agreement (−4.6–1.6 ± 3.7–$4.2^{\circ }$) were actually similar to previously reported marker-based error values [17].

In the context of fall risk assessment, 2D cameras have been used to monitor the gait in the elderly population. In a study by Wang et al. (2021) [7], a single RGB camera was used to assess gait parameters that could predict falls in older adults. The results of their study indicated that certain gait patterns (e.g., increased stride variability) were associated with an increased risk of falls. The use of two-dimensional video cameras allowed for continuous and unobtrusive monitoring of subjects, facilitating the early detection of fall risk and timely implementation of interventions.

While 2D camera systems offer a number of advantages, they are not without limitations. One such limitation is the lack of depth information and sensitivity to lighting conditions [17]. However, recent advances in image processing and machine learning have led to improvements in the accuracy and reliability of two-dimensional gait analysis systems. In addition, researchers are investigating the possibility of incorporating multiple two-dimensional cameras to obtain data from different perspectives, allowing for more comprehensive gait analysis [34].

#### 3.1.2. 3D Camera

The advent of 3D cameras has facilitated novel avenues for the analysis of human gait, which can be employed in a number of different fields, including clinical diagnosis, rehabilitation, sports biomechanics, and fall risk assessment. This is primarily attributable to the considerable advancement of visual sensor technology, machine learning algorithms, and computer processing capabilities, which enable more precise and comprehensive analysis of human gait movements.

In related research, Hatamzadeh et al. (2022) put forth a novel kinematic geometric model for spatiotemporal gait analysis using RGB-D cameras. They used a single Microsoft Azure Kinect camera to collect data, placing the camera 1 m from the end of the walkway and 80 cm in height. The model is based on the depth trajectories of the ankles and is enhanced by the application of sophisticated data processing techniques, thereby improving the accuracy of the results [5]. This innovation provides a portable markerless solution and also facilitates the use of three-dimensional cameras in clinical settings.

Moreover, Kobsar et al. (2019) investigated the potential of the Microsoft Kinect v2 depth camera in quantifying vertical oscillations in a running gait [35]. This study lends support to the proposition that Kinect v2 represents a viable and cost-effective alternative to traditional multi-camera motion capture systems, exhibiting accuracy in the capture of subtle movements that are pivotal for the assessment of running biomechanics. The cost-effectiveness and accuracy of Kinect v2 render it a particularly valuable tool in clinical settings, where budgetary constraints frequently restrict access to sophisticated technologies.

The innovation of depth camera technology is further demonstrated in the work of Díaz San Martín et al. (2021), who focused on using an integrated RGB and depth camera to enhance ankle joint angle measurement. Through using a region-based convolutional neural network (Mask R-CNN), the researchers were able to achieve accurate automatic ankle joint angle measurement, even when the lower limb was partially occluded from view [27]. Validation with an inertial measurement unit (IMU) showed a strong correlation and an acceptable error range, highlighting the potential of advanced algorithms to improve lower limb tracking accuracy in gait analysis.

Despite these improvements, challenges such as occlusion and tracking issues remain, necessitating continued research into enhancement algorithms and standardized protocols for different camera systems. In summary, although 3D cameras have made significant progress in gait analysis, continued innovation and interdisciplinary collaboration are essential to realize their full potential in clinical practice and beyond.

#### 3.1.3. Multi-Camera System

Multi-camera systems (including those produced by Vicon, OptiTrack, Qualisys, and Motion Analysis) are of significant value in the field of human gait analysis. These systems employ multiple high-speed cameras and corresponding software to capture and analyze human gait movements with great precision. Such systems are employed extensively across a variety of fields, including research, clinical settings, sports science, and the entertainment industry. This is due to their capacity to facilitate comprehensive three-dimensional reconstructions and insights into the biomechanics and movement control of the human body.

Of the aforementioned systems, Vicon’s multi-camera motion capture system is arguably one of the most widely used and applied. Ammann et al. (2020) employed Vicon’s 3D gait analysis to assess the postoperative outcomes of patients with patellar instability, thereby substantiating its efficacy in quantifying knee flexion angles and extensor moments, which are pivotal for surgical evaluation and treatment efficacy assessment [36].

Moreover, the Qualisys multi-camera motion capture system is a widely used system in the field. Sinsurin et al. (2020) employed this system to examine the biomechanical distinctions between ambulation and step-down activities. The researchers emphasized the pivotal function of the gluteus medius muscle in ensuring stability during movement, particularly in the context of functional tasks and rehabilitation strategies [37]. Similarly, Tanpure et al. (2023) employed Qualisys Oqus cameras for the purpose of conducting pre- and post-operative gait analysis on patients undergoing total knee arthroplasty (TKA). The findings revealed notable enhancements in gait parameters and joint kinematics following surgical intervention, thereby substantiating the efficacy of the Qualisys system in assessing surgical outcomes and formulating rehabilitation protocols [38].

Furthermore, Menascu et al. (2024) utilized the six-camera CODA 3D motion analysis system to investigate the joint angles exhibited during ambulation in young individuals diagnosed with multiple sclerosis (MS) and their healthy counterparts [20]. A reduction in the hip range of motion and knee flexion was observed, findings that could inform the development of clinical assessment tools and targeted interventions to enhance mobility and quality of life in young people with MS.

In conclusion, multi-camera systems are an indispensable tool for advancing human gait analysis as they provide precise and detailed motion data that are essential for clinical assessment, rehabilitation planning, and enhancing the understanding of biomechanical principles in various populations and functional tasks.

#### 3.1.4. Other Camera Systems

In addition to the commonly used 3D cameras, 2D cameras, and multi-camera systems, there are a number of vision-based systems that have been used with some success in human gait analysis. This section provides a summary of these vision systems.

In a recent paper, Duncan et al. presented a novel camera-based device for the monitoring and assessment of multiple physical fitness tests. The device was automated using a Raspberry Pi computer, three cameras, and two DC motors to facilitate the measurement and calculation of SPPB parameters [24]. The system used channel and spatial reliability tracking in the Python cv2 module and achieved high levels of accuracy, with over 95% for gait speed, across study groups comprising diverse volunteer populations.

Kaur et al. (2023) achieved high accuracy using a multi-view digital camera and deep learning algorithms to predict gait dysfunction in patients with multiple sclerosis and Parkinson’s disease, demonstrating the potential for cost-effective diagnosis of neurological disorders [39]. Similarly, Aung et al. (2020) validated the effectiveness of a video-based system against an instrumented gait system in patients who had suffered a stroke. The results showed that the system was able to accurately measure spatio-temporal gait parameters, even when tested by a novice [28]. The above studies demonstrate the practicality and accuracy of vision-based technology in the context of clinical gait assessment and rehabilitation.

Vats et al. (2022) explored the potential of smartphones for markerless gait analysis, with a particular focus on the effect of varying clothing on knee joint angle trajectories [40]. The researchers used an OpenPose-based methodology and a conventional smartphone camera to assess the influence of traditional Indian and regular clothing on the detection of knee joint coordinates. The results revealed significant discrepancies in the measurement of knee joint angles due to the influence of different types of clothing.

Taken together, these studies demonstrate the versatility and potential of various vision-based sensor systems for gait analysis. These systems offer practical and cost-effective solutions for monitoring and diagnosing gait abnormalities in a variety of populations, including elderly people with cancer, people with neurological disorders and stroke survivors. However, issues such as the impact of clothing changes and the accuracy of markerless systems need to be addressed to fully realize the potential of these systems in clinical settings.

### 3.2. Algorithms for Human Gait Analysis

To accurately analyze the human gait, it is not enough to rely on reliable visual sensor hardware. The implementation of robust algorithms for data extraction and analysis is also of paramount importance. This section provides an overview of the most commonly used machine learning algorithms in the field of human gait analysis. For example, nine studies, such as Sikandar’s, mainly used the LSTM (long short-term memory) networks algorithm; 12 studies, including Jamee’s, used CNNs (convolutional neural networks); three studies, such as Tasjid’s, used SVMs (Support Vector Machines); 22 studies, such as Sato’s, focused on pose estimation algorithms; and three studies, including Moghimifar’s, used the DTW (Dynamic Time Warping) algorithm.

#### 3.2.1. Long Short-Term Memory (LSTM) Networks

The long short-term memory (LSTM) algorithm is particularly well-suited for processing and predicting significant events in time series with extensive intervals and delays. Sivaprakash et al. (2023) employed a bidirectional LSTM to model the time-relatedness in pedestrian motion, thereby enabling the detection of anomalies during walking, including falls [41]. Similarly, Sikandar et al. (2021) employed a bidirectional LSTM to categorize walking speed based on two-dimensional image sequences, attaining a classification accuracy of 88.08% and 79.18% in indoor and outdoor settings, respectively. This technique does not necessitate the utilization of wearable devices and has been shown to be effective in both a clinical and an elderly care setting [32]. The aforementioned studies demonstrate the considerable advantage of LSTM in detecting minor temporal inconsistencies and identifying irregular gait patterns.

#### 3.2.2. Convolutional Neural Networks (CNNs)

In 2023, Vuong and Tran employed the use of ResNet Video R3D-18, a 3D convolutional neural network (CNN) method, with the objective of enhancing gait retrieval and identifying individuals based on their walking patterns. Their study demonstrated the efficacy of this method in capturing both spatial and temporal features from gait data, thereby addressing the challenges posed by subtle changes in contours. On the CASIA-B dataset, the authors achieved high accuracies of 97.09% for Rank-1 and 99.27% for Rank-10 under normal walking conditions [42].

#### 3.2.3. Convolutional Neural Networks (CNN) and Long Short-Term Memory (LSTM) Networks

Jaén-Vargas et al. (2021) conducted an investigation into the utilization of deep learning models for the purpose of human activity recognition (HAR), with a specific emphasis on the CNN-LSTM hybrid framework [43]. Their study integrated sensor data from inertial measurement units (IMUs) and a motion capture system (MOCAP) for the purpose of classifying activities such as walking, sit-to-stand, and squatting. The CNN-LSTM model demonstrated high accuracy, reaching up to 99%, which illustrated its effectiveness in integrating temporal and spatial data from diverse sensor types.

#### 3.2.4. Support Vector Machines (SVMs)

SVM algorithms are capable of effectively classifying individual gait patterns for identification purposes, of detecting abnormal gait behavior for the purposes of medical diagnosis, and of selecting relevant features from high-dimensional gait data. Chen et al. (2022) presented evidence of the effectiveness of SVM in conjunction with other machine learning methods for a range of gait classifications [12]. In terms of predicting falls on level ground, SVM achieved an accuracy rate of 94.9 ± 3.36%, which is considered to be impressive. Moreover, Alfayeed and Saini (2021) emphasized the pivotal role of SVM in discerning neurological effects, asymmetries, and gait disorders, as well as its versatility for clinical diagnosis and rehabilitation monitoring [11]. These studies reinforce the reliability of SVM and its substantial influence on the advancement of automated gait analysis systems.

#### 3.2.5. Pose Estimation Algorithms

In the field of human gait analysis, a dedicated algorithm has been employed for the purpose of extracting pivotal points of human posture. These include OpenPose for the detection of two-dimensional key points and skeletons; HRNet for the estimation of key points with high resolution and accuracy; DeepLabCut for the accurate tracking of key points in videos; PoseNet for the lightweight real-time estimation of two-dimensional poses; AlphaPose and DensePose for the mapping of body pixels to three-dimensional models; and MediaPipePose for the efficient detection of two-dimensional and three-dimensional key points. The aforementioned algorithms facilitate precise gait tracking and analysis, enabling the identification of abnormalities, the detection of atypical gait patterns, and the examination of behavioral patterns.

The potential and challenges of utilizing OpenPose for gait analysis are elucidated in recent research. Ino et al. (2023) validated AI-based gait analysis with a single camera, reporting mean absolute errors (MAE) of 2.3 to 3.1° on the camera side and 3.1 to 4.1° on the opposite side [15]. The coefficient of multiple correlation (CMC) indicated a high degree of waveform similarity, suggesting strong clinical applicability despite some accuracy issues. Nishizaki et al. (2021) developed a gait analysis tool utilizing OpenPose, which generates skeletal images over gait videos. This tool assists therapists in objectively detecting gait abnormalities and improving treatment in clinical rehabilitation [44].

#### 3.2.6. Dynamic Time Warping (DTW)

The dynamic time warping (DTW) algorithm is capable of accurately aligning and comparing gait sequences, even in the presence of differences in time or speed. The algorithm is capable of accurately measuring the similarity between disparate gait patterns, rendering it an optimal choice for identification, the detection of aberrant or pathological gaits, and the monitoring of rehabilitation progress. Furthermore, DTW is capable of accommodating alterations in walking velocity and stride length.

In a recent study, Talaa et al. (2023) introduced a video-based markerless system for automated home-based rehabilitation monitoring. The system is designed to accurately measure knee joint angles with a mean absolute error (MAE) of between 4.8° and 5.9° [1]. Moreover, Pattanapisont et al. (2023) explored the potential of gait pattern matching based on body angles calculated from skeletal joint coordinates. They employed the dynamic time warping (DTW) technique to assess the similarity of these patterns under diverse walking conditions and in response to changes in clothing [45]. These findings offer valuable insights for the enhancement of computer-based gait analysis.

### 3.3. Gait Parameter Analysis

**Figure 5:**
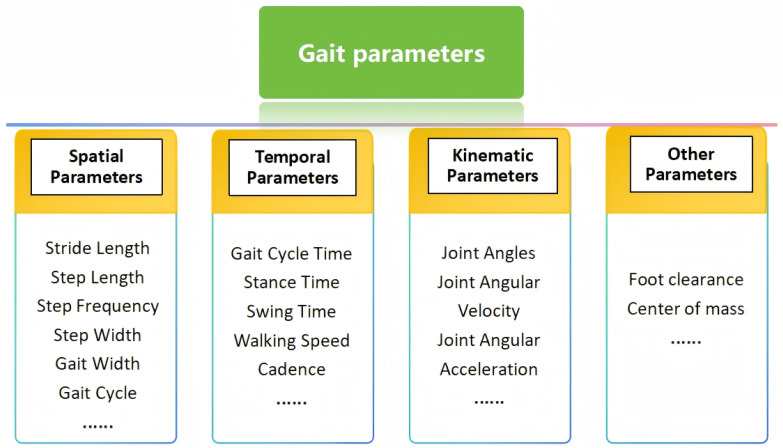
Categories of gait parameters in vision-based analysis.

The analysis of gait parameters is crucial in human gait analysis. By evaluating parameters such as stride length, stride frequency, and gait cycle, it is possible to effectively diagnose movement disorders, monitor the rehabilitation process, and optimize athletic performance. These parameters provide key data to identify potential problems in the nervous system or musculoskeletal system, and they also help athletes improve their technique and overall athletic performance. As shown in Figure 5, these parameters can be broadly categorized into spatial, temporal, and kinematic.

#### 3.3.1. Spatial Parameters

The analysis of spatial parameters in gait provides crucial insights into the morphology and stability of gaits, facilitating the identification of aberrant gait characteristics, the assessment of balance and coordination, and the detection of potential motor disorders or diseases. The term “step length” is defined as the distance between the successive placements of the same foot. This is measured by tracking the positions of the feet during the period of ground contact. Sensors or cameras are used to capture the heel strikes, which are then used to calculate the distance [23]. The distance from the initial contact of one foot to its next contact, defined as stride length, is determined by recording the entire walking cycle. The step width, defined as the lateral distance between the feet, is measured by recording the horizontal distance between foot centers [46]. This is achieved through the use of pressure sensors or vision-based systems, which are employed to assess balance and stability.

#### 3.3.2. Temporal Parameters

Cadence, defined as the number of steps per minute, is measured using vision-based systems that track foot movement in real time [47]. These systems quantify the number of steps within a specified interval by analyzing foot trajectories, thereby providing insight into walking speed and rhythm. Gait cycle time, defined as the interval between consecutive foot contacts, is measured by vision systems that detect the precise moments a foot strikes the ground and record the time until the next contact [45]. This allows for a comprehensive view of the various phases of walking, including stance and swing.

Stance time, defined as the duration a foot remains in contact with the ground, is quantified by vision sensors that monitor the moment the foot makes contact and subsequently lifts off [25]. Swing time [48], the time a foot spends in the air, is measured by capturing the foot’s motion as it leaves the ground until its next contact. Double support time [47], when both feet are on the ground simultaneously, is measured by detecting when both feet strike the ground. Vision-based systems track this period, providing valuable information about gait stability and balance.

#### 3.3.3. Kinematic Parameters

Kinematic parameters are critical for detailed analyses of the human gait, providing insights into joint motion and body posture throughout the walking cycle. Joint angles [49], such as those of the hip, knee, and ankle, are measured by vision-based systems that track the movement of key body points during walking. These systems use markers or advanced algorithms to calculate the angles formed at each joint in real time, allowing for analysis of the range of motion and detection of abnormalities like joint stiffness or asymmetry.

Pelvic tilt [50], the angle of the pelvis relative to the horizontal plane, is measured by tracking the position of the pelvis throughout the gait cycle. Vision sensors capture the pelvis’s movement, enabling precise measurement of its tilt and any deviations that might indicate compensatory mechanisms or gait issues. Trunk tilt [37], describing the inclination of the upper body, is extracted by monitoring the alignment of the torso as it moves during walking. Vision-based systems track the trunk’s movement relative to the vertical axis, providing insights into how the body maintains balance and compensates for lower limb issues. These kinematic parameters are measured through continuous motion tracking, providing valuable data for analyzing gait mechanics in clinical assessments, rehabilitation, and sports biomechanics.

#### 3.3.4. Other Parameters

Other parameters in gait analysis provide additional critical insights into various aspects of human locomotion beyond spatial, temporal, and kinematic measurements. Velocity [51], the overall walking speed, is calculated by combining cadence and stride length. Vision-based systems track foot movements in real time, measuring the distance covered and the time taken for each step. By analyzing this data, the system computes walking velocity, which is crucial for assessing walking efficiency and rehabilitation progress.

Center of mass (CoM) displacement [31] refers to the movement of the body’s mass relative to its base of support. Vision-based systems capture the position of key body points throughout the gait cycle, allowing for the tracking of CoM movements. These data help assess dynamic stability and balance control during walking, and deviations from normal patterns can signal potential fall risks or balance issues.

Foot clearance [52], the minimum height of the foot above the ground during the swing phase, is measured by vision systems that track the foot’s motion in detail. These systems monitor the foot’s trajectory to determine the exact height it reaches during the swing phase, which is essential for identifying risks of tripping or stumbling over obstacles.

Dynamic parameters such as joint moments [53], joint contact forces [26], centers of pressure [13], and ground reaction forces are of great importance. Joint moments reflect the rotational forces exerted on the joints and help assess the loading on muscles and joints; joint contact forces reveal the pressure distribution between joint surfaces, which affects the health of the joints; the center of pressure reflects the stability of the gait, and an abnormal COP trajectory may be indicative of an unstable gait or neuromuscular problems; and the ground reaction forces provide information on the mechanics of the body’s contact with the ground during gait, which helps analyze the gait thrust, braking force, and stability of the gait. These dynamic parameters are essential for the diagnosis of gait abnormalities, rehabilitation, and fall risk assessment.

These parameters, extracted through continuous tracking of body and foot movements via vision-based sensors, provide a comprehensive view of gait dynamics, contributing to improved assessments in clinical, rehabilitation, and performance settings.

### 3.4. Data Processing and Feature Extraction

In the field of vision-based gait analysis, the accurate processing and extraction of data are of paramount importance for the analysis of human movement patterns. These processes guarantee the extraction of pertinent parameters from video data, thereby facilitating a comprehensive understanding of gait dynamics. The following steps delineate the principal processes involved in transforming raw visual data into meaningful gait metrics.

- Preprocessing: Preprocessing in vision-based gait analysis involves noise reduction [5], image correction [54], and background subtraction [55] to refine raw data. These steps ensure accurate feature extraction and parameter calculation for detailed gait assessment and biomechanical analysis.
- Segmentation and Tracking: Segmentation and tracking in vision-based gait analysis employ advanced algorithms like OpenPose [15] or DeepLabCut to identify and continuously track the subject across video frames [44]. These methods utilize keypoints and pose estimation to ensure precise motion tracking, enabling detailed analyses of gait dynamics and biomechanical parameters with high accuracy and reliability.
- Feature Extraction: Feature extraction in gait analysis involves capturing joint coordinates [43], body segment lengths, and angles. These data points provide crucial insights into human movement patterns, aiding in clinical diagnostics and biomechanical research to enhance mobility and gait function assessment [40].
- Parameter Calculation: Parameter calculation in gait analysis uses extracted features to compute spatial (e.g., step length) [28], temporal (e.g., cadence), and kinematic parameters (e.g., joint angles). These metrics offer insights into human locomotion for clinical assessments and rehabilitation, optimizing interventions to improve movement efficiency and quality of life [27].

By following these steps, vision-based gait analysis systems can effectively process video data to extract detailed and reliable gait parameters. This workflow plays a critical role in various applications, from clinical diagnostics to rehabilitation planning, offering insights that help improve mobility, assess walking efficiency, and enhance the quality of life for individuals.

### 3.5. Number of Participants and the Experiment

In gait analysis, the experimental design and number of participants have a direct impact on the reliability and validity of the results. An adequate and diverse sample of participants ensures that the data are representative, covering different ages, genders, and health conditions, thereby improving the universality and accuracy of the analysis. In addition, an appropriate experimental design and sufficient sample size can improve the power of statistical analysis, reduce random error, and make the results of gait research more scientific and reproducible. Table 5 provides a summary of the experimental parameters and characteristics of several representative articles.

In their study, Keller et al. (2022) employed a sample of 29 healthy adults, thereby establishing a robust baseline for normal gait patterns. This approach allows researchers to establish a comparative standard against which abnormal gait behaviours can be measured [26]. In contrast, the Biomedical study (Biomedical, 2024) focused on 24 individuals, including those with movement disorders such as Parkinson’s disease, thereby underscoring the system’s potential for clinical use [56]. By including participants with specific health conditions, this study offers valuable insights into the potential of vision-based technologies for diagnosing and monitoring movement disorders. In summary, the integration of diverse participant cohorts and methodologies enhances the understanding of human gait analysis.

### 3.6. Camera Location

In experiments utilizing gait analysis, the positional distribution of the visual system is of paramount importance to ensure the accuracy of the data and the reliability of the ensuing analysis. The arrangement of visual sensors at varying angles and heights enables the capture of diverse aspects of gait, including the step length in the front view, the joint angle changes in the side view, and the gait trajectory in the top view. The reasonable distribution of the visual system’s positions can facilitate the comprehensive acquisition of spatial and temporal information about gait, minimize the presence of blind spots and data bias, and thus enhance the accuracy and credibility of gait analysis.

#### 3.6.1. Mobile Robot

The installation of a visual sensor on a mobile robot confers enhanced flexibility and dynamism, enabling the target to be pursued in real time and facilitating the acquisition of a more natural gait data. This approach also permits the acquisition of data from a broader field of view, thereby facilitating the collection of information from multiple angles. Furthermore, it enhances the comprehensiveness and accuracy of gait analysis.

Guffanti et al. (2022) demonstrated the potential of using robot-mounted 3D cameras for remote gait assessment, addressing the inherent limitations in 3D camera accuracy. Their study enhanced the kinematic and temporal estimates derived from a 3D camera by employing supervised learning techniques, specifically an artificial neural network that was trained using reference data from the Vicon system [60]. The integration of ANN-based optimization with a robot-mounted 3D camera not only improves the accuracy of gait data, but also demonstrates the flexibility and adaptability of this setup for analyzing gait impairments in natural environments.

Wang et al. (2021) installed an RGB camera on a mobile teleoperated robot, employing it to create a novel gait analysis method in response to the increasing necessity for health monitoring devices capable of executing reliable algorithms within a domestic setting. Their study effectively categorized walking patterns into distinct categories, including normal, supination, pronation, and limp [7]. This approach offers a cost-effective solution and demonstrates competitive performance in resource-constrained environments (e.g., those using a single entry-level CPU) when compared to more expensive multi-camera motion capture systems.

#### 3.6.2. Laboratory Environment

The laboratory setting offers the advantage of a controllable environment and high precision, which can ensure data stability and repeatability. Furthermore, through optimal arrangement, multi-angle coverage can be achieved. However, this fixed configuration, which lacks flexibility, may restrict the natural range of motion of the subject, and the results in the data are of a reduced applicability in real-world settings.

As demonstrated by Ammann et al. (2020), the utilization of a 3D gait analysis system comprising 12 VICON cameras and force plates enables the precise capture of gait kinematics and kinetics, which is pivotal for the assessment of the impact of trochanteric osteotomy on knee flexion and extension moments [36]. Similarly, Ripic et al. (2022) observed that an optoelectronic system comprising eight cameras and a force plate in conjunction with an Azure Kinect sensor exhibited a high correlation for ground reaction forces and joint moments [53]. This underscores the significance of a meticulously designed laboratory configuration for the acquisition of dependable kinetic data.

#### 3.6.3. Fixed in One Position

In their 2020 study, Yagi et al. proposed a home-based gait measurement system utilizing a single RGB camera, emphasizing the significance of camera placement [61]. In contrast to alternative measurement techniques that necessitate the use of multiple cameras or depth sensors, this system enables users to position a single camera within the designated area and identify four pre-defined points on the floor, thereby streamlining the configuration and capturing the gait position and time without the need for wearable devices. The system’s ease of use and high accuracy render it a practical solution for regular home health monitoring.

In a related study, Li et al. (2024) investigated the influence of camera positioning on gait recognition, utilizing drones to capture elevated vertical perspectives. The authors introduced the UAV gait dataset, which contains 22,000 sequences from 96 subjects with view angles ranging from 0° to 80° [62]. This highlights the challenges and opportunities of high vertical perspective recognition. The vertical refinement method can refine features from different vertical perspectives, which is significantly more effective than existing models. This demonstrates the importance of camera placement in improving gait recognition accuracy.

### 3.7. Applications of Vision-Based Gait Analysis

Vision-based gait analysis has been demonstrated to be a highly versatile tool with a broad range of applications across multiple domains, including sports performance, healthcare, home monitoring, the classification of walking speed, and the assessment of fall risk. By employing sophisticated computer vision methodologies, these systems offer non-invasive and precise analysis of human movement patterns, enabling the early identification of irregularities, performance enhancement, and rehabilitation.

#### 3.7.1. Sports Performance Analysis

In the field of sports biomechanics, vision-based gait analysis is employed with the objective of optimizing athletic performance and preventing injury. Systems that employ two-dimensional (2D) and three-dimensional (3D) cameras are able to track the complex movements of athletes during activities such as running, walking, and other dynamic movements. This allows for the analysis of essential biomechanical parameters, including joint angles, step length, and foot clearance [54]. The data are utilized by coaches and sports scientists to analyze posture, technique, and body alignment, thereby assisting athletes in enhancing their performance and reducing the risk of injury [35]. To illustrate, depth sensor-based systems, exemplified by Microsoft Kinect, have been employed to quantify vertical oscillation during running, thereby enabling the refinement of running posture to enhance efficiency and mitigate the risk of injury.

The knee is the runner’s most susceptible body part, with common injuries including anterior knee pain, iliotibial band friction syndrome (ITBS), and meniscus damage. For example, excessive adduction of the hip joint has been identified as a risk factor for iliotibial band friction syndrome (ITBS) and knee pain. An increased valgus angle and range of motion at the ankle joint have been identified as risk factors for running-related injuries. The aforementioned conditions can be quantified with the assistance of visual sensors.

#### 3.7.2. Healthcare and Clinical Diagnostics

In the field of healthcare, vision-based gait analysis is a widely employed method for the diagnosis and monitoring of gait abnormalities in patients with neurological disorders, musculoskeletal conditions, and those undergoing post-surgical rehabilitation [63]. For instance, two-dimensional (2D) and three-dimensional (3D) camera systems are employed to monitor patients’ gait patterns, with the objective of identifying abnormalities such as asymmetry, stiffness, or instability. These abnormalities may serve as indicative markers for conditions such as Parkinson’s disease, multiple sclerosis, Angelman Syndrome (AS), or the consequences of a stroke.

The aforementioned diseases are characterized by specific gait patterns. In Parkinson’s disease, for instance, patients typically exhibit muscle stiffness, slow movement, stooping, slow gait, and a reduced stride length. As the disease progresses, patients may develop gait disorders, which are characterized by a gradual increase in stride speed and a reduction in stride length, significantly elevating the risk of falls. The gait in patients with MS is typically characterized by reduced speed, impaired balance, and an increased time spent on both feet. Patients tend to utilize a wider support surface in order to maintain stability, and these gait changes are influenced by the fear of falling. Stroke survivors frequently experience gait disorders, including decreased muscle strength, decreased coordination, and balance problems. Such patients often exhibit spasticity, which can result in decreased balance and gait asymmetry. The gait of individuals with Angel Syndrome (AS) is characterized by severe movement disorders, including movement and balance difficulties. Those with AS display poor coordination, unsteady movements, and difficulty maintaining balance, which can impact their overall gait and mobility.

The analysis and identification of these disorders can be facilitated by the use of visual sensor systems, which allow for the observation and measurement of gait characteristics. Furthermore, these systems are vital for evaluating the efficacy of rehabilitation programs as they monitor the advancement in gait parameters over time [32]. Gait analysis enables clinicians to devise bespoke treatment plans that enhance mobility and improve patient prognosis.

#### 3.7.3. Home Monitoring and Elderly Care

The use of vision-based gait analysis systems is becoming increasingly prevalent in the field of domestic monitoring, particularly in the context of elderly care. Systems equipped with a two-dimensional camera or depth sensors are capable of monitoring walking patterns in real time and without interference, providing information such as gait speed, balance, and foot clearance [64]. Such systems have been demonstrated to be highly effective in the early detection of gait deterioration or an increased risk of falls, thereby facilitating timely intervention. For instance, research has demonstrated that gait monitoring systems in elderly care can predict fall risk based on specific gait parameters, including step length variability and double support time. This allows caregivers and healthcare professionals to utilize actionable data in order to prevent falls [7].

#### 3.7.4. Walking Speed Classification

The classification of walking speed represents a further key application of visual gait analysis. By capturing comprehensive data on foot and joint movements, these systems are able to categorize walking speed as slow, normal, or fast. This classification can be employed in a clinical setting to evaluate a patient’s mobility status, particularly in cases of surgical or traumatic injury, where walking speed can serve as an indicator of functional recovery. For instance, studies have employed two-dimensional camera systems with deep learning algorithms, such as long short-term memory (LSTM) networks, to accurately categorize walking speed based on image sequences. This demonstrates the potential of such systems in clinical diagnosis and rehabilitation [32].

#### 3.7.5. Fall Risk Assessment

Vision-based systems are extensively employed for the assessment of fall risk, particularly in elderly populations or patients with neurological disorders. By continuously monitoring gait patterns, these systems are able to identify key gait parameters, including stride variability, step width, and foot clearance, which are predictive of fall risk [12]. The integration of machine learning algorithms with vision-based systems enables the real-time assessment of fall risk, facilitating prompt interventions and reducing the probability of falls. For example, vision-based systems have been incorporated into elderly care settings to observe gait patterns and issue timely alerts regarding fall risks, thereby enhancing safety and improving the quality of life of the elderly [31].

#### 3.7.6. Rehabilitation and Mobility Enhancement

In the context of rehabilitation, vision-based gait analysis plays a pivotal role in monitoring patient progress and facilitating the adaptation of treatment plans. Such systems permit therapists to assess alterations in gait parameters throughout the course of rehabilitation programs, particularly in patients recuperating from surgical procedures such as total knee arthroplasty (TKA) or hip replacements [14]. The provision of real-time feedback on gait dynamics enables the optimization of therapy sessions and the monitoring of improvements.

## 4. Discussion

In this review, we provide a comprehensive overview of the latest advances in vision sensor-based human gait analysis systems between 2019 and 2024, with a special emphasis on the role of vision sensors, machine learning algorithms, and technological innovations. The integration of these cutting-edge technologies has significantly improved the accuracy and efficiency of gait analysis systems, making them increasingly important in several domains (e.g., healthcare, sports science, and security).

Despite significant progress in the field, a number of challenges and limitations remain, especially in the practical application of these technologies. Continuous improvements in hardware, software, and algorithm design are crucial, but addressing practical barriers such as data quality, sensor accuracy, and computational requirements remains important for the further development of gait analysis systems. This discussion section will delve into these key findings, analyze the impact of the progress made in the previous sections, and identify gaps and opportunities for future research.

### 4.1. Vision-Based Sensors

Visual sensor systems employed in the field of human gait analysis can be classified into a number of distinct categories. These include RGB cameras, depth sensors such as Kinect, marker-based motion capture systems, and markerless motion capture systems. Each type of sensor exhibits distinctive performance characteristics, frame rates, accuracy levels, strengths, limitations, and application scenarios.

Two-dimensional cameras are frequently employed for recording the gait of participants. The frame rates typically observed for these systems range from 30 to 120 frames per second (fps), depending on the camera model and settings [6]. These cameras are inexpensive, simple to install and use, and suitable for a variety of applications. However, the lack of depth information limits the scope of 3D analysis, and these devices are susceptible to light fluctuations and occlusions, which reduces their accuracy in correlating 2D data [13]. RGB cameras are frequently employed in clinical diagnostics, fall risk assessment, and fundamental motion analysis within controlled indoor settings, largely due to their affordability and simplicity. This also renders them suitable for preliminary screenings and educational purposes.

Depth sensors such as the Azure Kinect DK are capable of capturing both color (RGB) and depth information. They typically operate at frame rates of around 30 fps (frames per second), as evidenced by the cited references: [5,7,19,65]. These sensors provide depth data for accurate three-dimensional positioning and movement tracking; however, their accuracy can be affected by occlusions and reflective surfaces, and their performance may degrade in outdoor or brightly lit environments [64]. Although they have a restricted range and resolution in comparison to high-end systems, depth sensors provide three-dimensional depth data, thereby reducing the complexity and intrusiveness of the setup while maintaining effectiveness for joint angle measurements and three-dimensional motion tracking [4]. They are particularly well suited to clinical settings, including rehabilitation monitoring and precise joint angle measurements. They are also popular for in-home health assessments and physical therapy due to their balance of detailed analysis and ease of use.

Marker-based systems, such as Vicon or OptiTrack, utilize reflective markers attached to the body, which are tracked by multiple cameras in order to capture precise three-dimensional movements [1]. These systems operate at high frame rates, often exceeding 100 fps, thereby providing extremely accurate and detailed biomechanical data [66]. They are highly precise and robust against environmental factors such as lighting and occlusions, thereby establishing them as the gold standard for research and detailed biomechanical analysis [29]. However, they are costly and intricate to configure and operate, and the necessity of attaching markers to the body can impede natural movement, necessitating a controlled environment for optimal performance [46]. Marker-based systems are the preferred choice in research and clinical settings where high accuracy is essential, such as in detailed biomechanical studies, sports science research, and advanced clinical diagnostics. Despite the logistical challenges and high costs associated with these systems, they remain the gold standard for research and detailed biomechanical analyses.

Markerless motion capture systems employ sophisticated algorithms and multiple cameras to track movement without the necessity for physical markers, typically achieving frame rates of 30 to 60 fps [24]. As computer vision and machine learning continue to advance, these systems are becoming increasingly accurate, non-intrusive, and flexible, allowing for natural movement and adapting to various environments. This reduces setup time and complexity compared to marker-based systems [61]. However, there are some limitations to their accuracy in complex environments or with occlusions, and their performance can be affected by factors such as clothing and background textures. Although they are still evolving to match the precision of marker-based systems, markerless systems are gaining popularity for applications requiring natural movement, such as in sports, ergonomics, and some clinical settings, particularly where quick setup and flexibility are needed [67]. An example of this is the AI-powered multi-camera replay systems used in the Paris Olympics, which was developed by Alibaba. These systems facilitate real-time three-dimensional rendering and spatial reconstruction of athletes’ movements, thereby enabling the generation of immersive replays in a matter of seconds. Continued advances are being made that are enhancing the reliability and accuracy of these systems.

The future of visual sensor systems in human gait analysis will be characterized by significant advancements, including the integration of multiple sensor types and algorithm enhancements. The combination of depth sensors with RGB cameras will provide both detailed three-dimensional motion data and rich color information, thereby overcoming the limitations of each sensor type when used alone [67]. The use of multi-sensor networks, including distributed sensor arrays, will facilitate the coverage of larger areas and reduction in occlusion issues, thereby enabling gait analysis in more complex environments, including outdoor settings [68].

Further improvements in the capabilities of these systems will be achieved through algorithm enhancements based on machine learning and AI techniques [69]. The incorporation of advanced pose estimation algorithms will enhance the precision of markerless systems, even in challenging conditions characterized by occlusions and varying backgrounds. The application of predictive analytics will enable AI to anticipate potential gait abnormalities and provide timely alerts for clinical interventions, as outlined by Vats [40].

The integration of data from different sensors through data fusion techniques will enhance the robustness and accuracy of gait analysis, providing a more holistic view of a subject’s movement. The combination of multiple visual sensor types and the enhancement of algorithm capabilities through machine learning and AI will facilitate the development of gait analysis systems that are more accurate, versatile, and widely applicable. This integrative approach will improve the accuracy and applicability of these systems across diverse populations and clinical conditions, facilitating more effective human gait analysis in both research and clinical practice.

### 4.2. Optimal Measurement Position for Vision Sensors

In the field of human gait analysis utilizing vision sensor systems, the positioning of diverse visual sensors is of paramount importance for the acquisition of precise and comprehensive gait data. The optimal placement and measurement capabilities of each type of sensor—including RGB cameras, depth sensors, marker-based motion capture systems, and markerless motion capture systems—are contingent upon specific considerations.

Two-dimensional cameras are typically positioned in a strategic manner around the subject in order to capture two-dimensional color images and videos. Such cameras are typically positioned in front of the subject to record frontal views, laterally to capture lateral movements, and sometimes overhead to observe vertical dynamics [27]. Such cameras are particularly adept at capturing detailed visual information, including aspects such as body posture, stride length, and foot clearance. However, due to their lack of depth perception, they are better suited for basic motion analysis and qualitative assessments in controlled environments where lighting and occlusions can be managed.

Depth sensors, such as those found in Kinect devices, are positioned to encompass the entirety of the body’s potential range of motion in an effective manner [70]. Ideally positioned in a central location and at an appropriate height, these sensors provide precise three-dimensional spatial information, which is crucial for accurate joint angle measurements and detailed motion tracking [56]. The placement of these devices is designed to minimize occlusions and optimize depth data accuracy. However, it should be noted that environmental factors such as lighting and reflective surfaces can affect their performance, particularly in outdoor or complex settings. For example, the ZED 2 by Stereolabs is an advanced depth camera offering wide-angle depth sensing up to 20 m with a 120° field of view. Furthermore, the device is equipped with artificial intelligence (AI) processing capabilities, which facilitate object detection, spatial mapping, and simultaneous localization and mapping (SLAM). Additionally, the camera is capable of tracking multiple individuals simultaneously.

In marker-based motion capture systems, reflective markers are placed on key anatomical landmarks of the body. A number of high-speed cameras are positioned around the capture volume, capturing overlapping views in order to guarantee the continuous tracking of marker positions throughout the gait cycle. This configuration permits the precise measurement of joint angles, segmental movements, and overall biomechanical parameters [71]. The cameras are positioned in a way that minimizes occlusions and optimizes marker visibility, thereby ensuring high accuracy in three-dimensional motion analysis. One of the principal limitations of marker-based motion capture systems is the marker occlusion problem, whereby markers may be temporarily obscured from the camera’s view, particularly during complex movements or when multiple body parts are in close proximity. Furthermore, the system necessitates a controlled laboratory setting to minimize the impact of lighting interference and reflections, which may restrict its deployment in more dynamic environments. Furthermore, the necessity for a considerable number of markers entails a lengthy preparation period and the utilization of sophisticated apparatus and calibration processes, which constrains the accessibility and scalability of these systems for routine clinical applications [72].

Markerless motion capture systems (comprising any combination of multiple 2D or 3D cameras) employ sophisticated computer vision algorithms and synchronized cameras to track movement without the use of physical markers. These systems offer the flexibility to place sensors in a variety of locations, adapting to different environments and enabling the capture of natural movement dynamics during gait analysis. Cameras are positioned in a strategic manner to achieve comprehensive coverage and minimize occlusions, thereby allowing for unrestricted movement while maintaining accuracy in tracking joint positions and motion patterns [73]. The popularity of markerless systems is growing in the fields of sports science, clinical rehabilitation, and ergonomic assessments due to their non-invasive nature and ability to capture real-world movement scenarios.

In the future, optimizing sensor placement and measurement strategies across these visual sensor types will be crucial for advancing human gait analysis. The integration of complementary strengths, such as the use of RGB cameras for initial screening and markerless systems for detailed biomechanical analysis, promises to enhance overall accuracy and reliability [39]. Future developments in sensor technology and algorithmic sophistication will further mitigate environmental challenges and expand the applicability of visual sensor systems in diverse real-world settings.

### 4.3. Strengths, Weaknesses, and Applicability of Gait Analysis Algorithms

LSTM are particularly well suited to the analysis of time-series data, and thus to the capture of temporal changes that are critical for the detection of gait abnormalities such as falls [41]. The ability to learn long-term dependencies is a key advantage of LSTMs, making them well suited to detailed temporal analyses in clinical and geriatric care environments. In such settings, the use of wearable devices may be avoided, which is beneficial. However, LSTMs are computationally intensive, which presents a challenge for their implementation in real-time scenarios, particularly on devices with limited computational resources.

CNNs are particularly adept at extracting spatial features from gait data and are capable of efficiently recognizing individuals and anomalies based on walking patterns [42]. The primary advantage of CNNs is that they generalize well across different datasets, enabling the identification of complex spatial features with high accuracy. However, their efficacy is contingent upon the availability of extensive labeled datasets, which can be a constraint when such data are scarce or costly to procure. Convolutional neural networks (CNNs) are particularly well suited to scenarios necessitating detailed spatial analysis, such as identification and diagnostic assessments in clinical settings.

Hybrid CNN-LSTM networks employ the respective strengths of both CNN and LSTM to effectively capture both the spatial and temporal features from gait data. This dual capability permits a more comprehensive analysis of gait, rendering these hybrid models effective for detailed behavioral analysis and activity recognition [43]. However, the combination of CNNs and LSTMs markedly increases the complexity and computational demands of the models, thereby limiting their suitability for real-time applications and resource-constrained environments. Nevertheless, they are of value for comprehensive gait analysis tasks where both spatial and temporal insights are critical.

SVMs are a robust classifier for gait pattern recognition, exhibiting high accuracy in the detection of gait anomalies and the differentiation between various gait patterns [11]. In comparison to other machine learning models, SVMs have relatively low computational requirements, which makes them an effective choice for real-time applications. However, SVMs may encounter difficulties in addressing the complexity and scale of large datasets, as well as the variety and nuance of gait, which can impact their effectiveness in more complex gait scenarios. They are most appropriate for relatively small-scale and well-defined gait analysis tasks.

Pose estimation algorithms (e.g., OpenPose) offer a non-invasive method of extracting detailed skeletal information from video sequences, which is beneficial for precise gait analysis in clinical settings. The principal advantage of these techniques is the objective and precise acquisition of data, which does not require the use of physical markers [15]. This makes them an attractive option for clinical and rehabilitative purposes. However, the accuracy of these methods can be compromised by environmental factors, such as occlusion and changing light conditions, which can affect the reliability of the gait data obtained. Notwithstanding these challenges, posture estimation algorithms are well suited to environments where non-invasive detailed motion analysis is required.

DTW is a distance-based algorithm that measures the similarity between time-series data, rendering it an optimal choice for gait pattern matching and anomaly detection. This approach has the advantage of being able to accommodate variations in walking speed and body motion, which is essential for accurate comparison of gait cycles [45]. Nevertheless, DTW is a computationally intensive method, particularly when applied to large datasets, which restricts its use in real-time applications. It is most appropriate for offline analysis, where the objective is to identify similarities or anomalies in gait sequences, and where real-time computation is not a primary requirement.

Transformer-based models [64], diffusion-based models and reinforcement learning models are all cutting-edge technologies in recent years, but their applications are still in the exploratory stage. Transformer-based models, such as Vision Transformer-based models, e.g., Vision Transformer (ViT), are able to effectively capture long-term dependencies in gait sequences through the self-attention mechanism, and they are especially good at processing time-series data, which improves the accuracy of gait recognition. Diffusion-based models are mainly used for data augmentation and missing data reconstruction, which can generate high-quality gait data and enhance the diversity of training datasets, but their applications in gait recognition are still limited [70]. Reinforcement learning models are commonly used in gait rehabilitation and smart exoskeletons, which can dynamically optimize gait patterns based on user feedback, but their generality and effectiveness still need to be verified. Overall, these SOTA models have advantages in gait analysis, but they are still in the preliminary stage and have great potential for future research.

In conclusion, the selection of an algorithm for human gait analysis should be informed by the specific requirements of the intended application, including the necessity for real-time processing, the availability of data, and the availability of computational resources. LSTM networks are optimal for temporal pattern recognition [32], while CNNs and hybrid models are best suited for capturing both spatial and temporal features [11]. SVMs are most effective for smaller datasets, while pose estimation algorithms are best employed for detailed skeletal analysis. DTW is the most appropriate algorithm for pattern matching (Alfayeed, 2021). It is therefore crucial to understand these strengths and limitations in order to select the most appropriate algorithm for optimal performance in gait analysis tasks.

### 4.4. Comprehensive Analysis of Gait Parameters in Vision-Based Gait Analysis

In the field of vision-based gait analysis, a range of parameters are typically extracted with the objective of providing a comprehensive understanding of an individual’s walking patterns. These parameters can be classified into three domains: spatial, temporal, and kinematic. Each domain provides unique insights into different aspects of human locomotion.

Spatial parameters encompass measurements such as step length, stride length, and step width. The assessment of step length and stride length is of critical importance for the evaluation of gait stability and efficiency. These parameters reflect the distance traversed by an individual with each step and the total distance covered during a full gait cycle [23]. The measurement of step width provides insight into the individual’s ability to maintain balance and stability during gait. These parameters are of critical importance for a range of applications, including biomechanical research, clinical diagnostics, and rehabilitation. For example, depth cameras can be used to assess changes in stride length in patients with Parkinson’s disease, thereby facilitating a better understanding of their balance issues. Similarly, Kinect-based vision systems can be employed to assess stride length anomalies in post-stroke patients, enabling the tracking of recovery progress [46].

Temporal parameters, including cadence, gait cycle time, stance time, swing time, and double support time, provide detailed insights into the timing and rhythm of walking. Cadence, defined as the number of steps taken per minute, provides an indication of walking speed and rhythmicity. Gait cycle time, on the other hand, encompasses the duration of a complete gait cycle, comprising the stance and swing phases [45]. Stance time and swing time are indicative of the periods of weight bearing and foot movement, respectively, whereas double support time reflects the duration of simultaneous ground contact by both feet. These temporal metrics are of great importance for the evaluation of gait efficiency, performance, and stability, and they are widely employed in clinical assessments and rehabilitation strategies [48]. For instance, a video-based analysis system was utilized to observe the alterations in standing time during rehabilitation following ankle sprains, providing comprehensive feedback to physiotherapists to facilitate the personalization of treatment.

Kinematic parameters concentrate on joint angles, pelvic tilt, and trunk tilt, thus providing a comprehensive analysis of joint movement and body posture throughout the entire walking cycle. The range of motion and dynamics at the hip, knee, and ankle joints can be elucidated through the observation of joint angles, which can also serve to identify abnormalities such as joint stiffness or asymmetries [49]. The angles of the pelvis and trunk relative to the horizontal plane are measured by pelvic tilt and trunk tilt, respectively. These kinematic measurements are fundamental to the comprehension of human locomotion in the fields of sports biomechanics, clinical practice, and rehabilitation [37]. For instance, the utilization of vision-based systems, such as three-dimensional motion capture employing multiple camera configurations, has been employed to evaluate the performance of an athlete. The hip angle can be optimized for enhanced running or jumping performance by rectifying sub-optimal movement patterns. Similarly, the use of RGB-D cameras can facilitate the assessment of torso tilt compensation in patients with spinal cord injuries during rehabilitation, thereby assisting clinicians in adapting therapeutic strategies to improve posture and balance control.

Furthermore, additional parameters such as walking velocity, center of mass (CoM) displacement, and foot clearance contribute to the comprehensive analysis of gait patterns [31]. The velocity of walking, derived from the combination of cadence and stride length, represents a fundamental measure of walking efficiency and performance. The displacement of the center of mass (CoM) tracks the movement of the body’s mass relative to its base of support. This is a crucial factor in understanding dynamic stability and energy efficiency during walking [51]. The measurement of foot clearance is essential for the assessment of the risk of tripping and falling as it determines the minimum height of the foot above the ground during the swing phase. These additional parameters provide a comprehensive perspective on gait mechanics, complementing the spatial, temporal, and kinematic metrics.

Joint moments [53] are commonly used to identify joint loading problems in specific movement patterns to help develop individualized exercise intervention strategies; joint contact forces are used to assess joint surface pressure distributions, providing data to support joint protection and rehabilitation program optimization; center of pressure (COP) [13] trajectories are used to detect gait stability abnormalities, especially in neuromuscular disorders or fall risk prediction for the elderly; and Ground Reaction Force (GRF) helps to analyze gait dynamics characteristics by quantifying the thrust and braking forces of gait. The combined use of these parameters not only improves the accuracy of gait analysis, but it also provides a scientific basis for clinical intervention and rehabilitation strategies.

In conclusion, the most commonly used gait parameters in vision-based gait analysis are those that fall within the spatial, temporal, and kinematic domains. Each of these domains offers unique insights into the patterns of walking [25]. Spatial parameters assess stability and efficiency, temporal parameters evaluate timing and rhythm, and kinematic parameters analyze joint motion and posture [37]. Additional metrics such as velocity, center of mass displacement, and foot clearance provide further insights into gait efficiency, stability, and safety [47]. The integration of these parameters into gait assessments enables a comprehensive understanding of human locomotion, facilitating targeted interventions in clinical, biomechanical, and rehabilitative applications to enhance gait function, mitigate injury risks, and improve overall mobility and quality of life.

### 4.5. Review Limitations

It should be noted that this review article is subject to a number of limitations. Firstly, the review article in question places a significant emphasis on vision-based sensor technologies, which may result in the overlooking of notable contributions from other modalities, such as wearable sensors or hybrid systems that combine multiple sensor types. This review is limited in scope to articles published between 2019 and 2024, which may result in the exclusion of earlier influential research and developments in the field of gait analysis.

### 4.6. Existing Challenges and Future Works

#### 4.6.1. Existing Challenges

Despite the considerable advancements made in the domain of vision-based gait analysis, numerous challenges persist. A significant hurdle is the variability in environmental circumstances, encompassing factors such as lighting and occlusions, which can markedly influence the precision of visual sensor data. Furthermore, the efficacy of markerless motion capture systems may be constrained by variables including clothing alterations and background intricacy, impeding the precise tracking of bodily movements.

#### 4.6.2. Future Works

To address these challenges, future research could explore hybrid sensor systems that combine vision sensors with wearable inertial measurement units. This combination could improve accuracy and reliability, especially under changing conditions such as low light or crowded environments. In addition, improved methods for calibrating sensors for different environments could be effective in overcoming existing performance limitations.

In terms of algorithms, more advanced machine learning models, especially deep learning techniques such as convolutional neural networks (CNNs), are recommended to handle complex and noisy data and improve the accuracy of gait analysis. In addition, exploring transfer learning techniques can improve the ability of models to generalize across different environments and populations to address individual gait differences. Developing algorithms that are robust to these differences will be critical to achieving more reliable gait analysis results.

Camera placement remains a key issue. To improve the accuracy of gait tracking, researchers can try multi-camera setups with integrated depth cameras or 3D vision systems (e.g., LiDAR) to acquire gait data from multiple angles. In addition, combining ground and elevated camera views can reduce the occlusion problem and provide a more comprehensive gait analysis, especially in real-world applications when subjects are not always clearly captured by a single viewpoint.

Finally, more comparative studies are needed to evaluate the effectiveness of different combinations of sensors, algorithms, and camera placement. These studies will provide clearer guidelines for choosing the best approach in different gait analysis applications, such as in healthcare, sports science, or security. By focusing on these areas, future research can help address existing limitations and drive the development of more accurate and reliable vision-based gait analysis systems.

## 5. Conclusions

The field of human gait analysis has witnessed significant advancements with the emergence of vision-based sensor technologies, effectively transforming it from traditional, subjective assessments to highly precise, high-resolution methods. This review emphasizes the integration of RGB cameras, depth sensors, and markerless motion capture systems, demonstrating their capacity to capture comprehensive spatiotemporal data, which is vital for applications in healthcare, rehabilitation, sports science, and security.

This review categorized various visual sensors, discussed optimal placement strategies, and evaluated the algorithms, such as convolutional neural networks (CNNs), long short-term memory (LSTM) units, and support vector machines (SVMs), used in gait analysis. Furthermore, this review presents a comprehensive analysis of gait parameters across spatial, temporal, and kinematic domains, emphasizing the significance of these metrics in understanding human locomotion. Despite the notable advancements, several challenges remain, including the impact of environmental factors, the necessity for computational efficiency, and the demand for high-quality, diverse datasets. It is recommended that future research should focus on the development of robust algorithms that can handle variability, the integration of multimodal sensor systems, and the fostering of collaborations between researchers, clinicians, and industry practitioners.

By addressing these challenges and capitalizing on the strengths of vision-based technologies, the field can enhance the accuracy, robustness, and applicability of gait analysis systems. In conclusion, these developments will contribute to enhanced clinical outcomes, more efficacious rehabilitation protocols and a more profound comprehension of human movement, thus improving the quality of life across diverse populations.

## Figures and Tables

**Table 1:** Summary and differences of recently published review articles on vision-based sensors for human gait analysis.

Paper Title	Year	Model	Systematic Review	Publication Year Range of Studied Articles	Number of Studies Included	Differences with Respect to Our Article
[8]	2022	SVM, KNN, CNN, LSTM	Yes	2015–2022	128	Only some vision sensors are mentioned, but their functions are not described in detail
[9]	2021	Various traditional ML and DL models	Yes	2010–2021	204	No mention of 3D cameras
[10]	2023	Deep learning models (e.g., CNN, RNN)	Yes	2018–2023	29	Only 2D video and image analysis
[11]	2020	Machine learning models	Yes	2015–2020	36	Only 36 articles were analyzed, and the machine learning method is not comprehensive
[12]	2022	ML techniques (e.g., SVM, KNN)	Yes	2017–2022	89	Only limited to vision-based deep learning approaches

**Table 2:** Databases and queries for literature search.

Database	Query
IEEE Xplore	(“All Metadata”: camera* OR “All Metadata”: video*) AND (“All Metadata”: human gait*) AND (“All Metadata”: walk* OR “All Metadata”: run* OR “All Metadata”: jog*)
Scopus	(TITLE-ABS-KEY (camera* OR video*) AND TITLE-ABS-KEY (human AND gait*) AND TITLE-ABS-KEY (walk* OR run* OR jog*)) AND PUBYEAR &gt; 2018
Web of Science	camera* OR video* (Abstract) and run* OR walk* OR jog* (Abstract) and human gait* (Abstract)
PubMed	((camera* [Title/Abstract] OR video* [Title/Abstract]) AND (run* [Title/Abstract] OR walk* [Title/Abstract] OR jog* [Title/Abstract])) AND (human gait* [Title/Abstract]) Filters: from 2019–2024

Note: Adding “*” in search expands results by matching all words starting with the root.

**Table 3:** Inclusion and exclusion criteria.

Inclusion Criteria	Exclusion Criteria
Gait analysis systems using vision sensors.	Book chapters, review papers, letters, short communications, technical notes, conference proceedings.
Sensor modalities include 2D, 3D, ordinary, depth, multi-camera, and other vision-based systems.	Only evaluated vision sensor technology for step counts, distance, and activity classification.
Included at least one defined gait outcome measure, such as spatiotemporal or kinematics.	Studies not evaluating straight walking (e.g., changes in direction tasks or cutting manoeuvres)
Written in English.	Aimed to evaluate computer algorithms, machine learning, or statistical approaches.
Based on video recording and gait analysis.	Study concerns non-human animal subjects
Investigated gait variability or regularity.	Study involves clinical gait analysis only.
Open-access articles.	Studies involve wearable sensors, such as IMU, etc.

**Table 4:** Summary of the main contents of the reviewed articles.

Authors	Vision Sensors	Frame Rates	Location	Gait Parameters	Limitations and Further Study
Ceriola et al. [6]	A Logitech BRIO 4k Stream Edition camera	f (1920 × 1080 px) and 60 Hz.	The subject performed four trials of one-minute walking on a treadmill at three different speeds.	Stride length/stride time/step length/step width.	The 30 Hz sample rate limits Azure Kinect’s accuracy at faster speeds and for stance/swing time measurements.
Erika et al. [13]	Two identical high-definition webcams (Logitech brio 4k stream edition).	60 frames and 720 × 1280 pixel.	The webcams were placed in three different positions: back–back, lateral–lateral, and back–lateral.	Specific lower limb joint angle.	The system’s kinematic accuracy is limited by camera configuration and locomotion activity.
Peebles et al. [14]	A 10-camera motion capture system (Oqus 700, Qualisys, Goteborg, Sweden). A single consumer-grade video camera (Hero 6 Black, GoPro, San Mateo, CA, USA)	120 frames and an image size of 1920 × 9 × 1080 pixels.	The camera was placed at five positions, spanning angles of 35.9° to 0°.	Knee, ankle, and foot angle at contact, peak knee flexion, knee flexion excursion, and knee–ankle flexion vector coding variability.	The study’s limitations include marker identification variance, single-session design, and sagittal plane focus.
Ino et al. [15]	A high-speed digital video camera (Bonita Video 720C, Vicon Motion Systems Ltd., Oxford, UK).	120 H	The camera captured sagittal views from the participants right side to ensure accurate visualization of the motion.	Ankle joint, knee joint, and hip joint.	Study limitations include AI misidentification, inherent 3D-MA errors, and lack of validation in abnormal gait patterns.
Ho et al. [16]	ZED camera.	30 frames and a resolution of 1080 p.	Participants walked a 4 m line.	Stride length/stride time/step length/step width.	Study limitations include requiring a 3D camera and varying performance with different cameras.
Wade et al. [17]	Fifteen Qualisys cameras (Oqus, Qualysis, Gothenburg, Sweden) and two machine vision cameras.	200 Hz.	One machine vision camera was set up in the sagittal plane (right hand side of the body during walking) and one was set up in the frontal plane (directly in front of the participant).	Ankle, hip, and knee.	Limitations include marker-based errors, indoor settings, and a small, healthy adult participant group.
Vairis et al. [18]	Two iPhone 6s.	8 megapixel cameras and 30 frames per second.	With one recording the side view of the walk and the second camera, at 90° to the first camera, recording the subject walking toward the camera.		
Beatriz et al. [19]	Portable low-cost devices (KinecteMotion).	30 Hz.	The camera is placed in front of the participant.	Swing magnitude (left/right), swing time (left/right), swing speed (left/right), and arm swing asymmetry (ASA).	Study limitations include small sample size, single dataset, no gait speed matching, and unimplemented ML algorithms.
Menascu et al. [20]	A six-camera (CX1 sensor unit) CODA 3D motion analysis system.	4 s at 200 Hz	The camera is placed in front of the participant.	Walking speed, stride length, stride time, cadence (steps/min), step length, stance phase, and step time.	Limitations include a small sample size, retrospective design, unrecorded factors, and no control group.
Lee et al. [21]	four different cameras.		Each camera was placed three meters from the center of the pedestrian path, and the cameras at the front and back were about one meter from the end of the walkway.	Step length, walking speed, swing/stance phase, foot angle, knee angle, and knee varus/valgus.	Limitations include a lack of dominant hand data and potential manual measurement errors.
Lawrence et al. [22]	Video camera.	30 frames.	Physical layout shows the camera capturing a person’s gait as that person walks at 90° to the camera view.	Speed, variability, step duration, activity level, stride length, and multi-scale entropy.	Limitations include constrained system setup and challenges in achieving accurate gait measurement.
Giannakou et al. [23]	Six optoelectronic cameras and two Kistler force plates.	100 fr/s and 1000 Hz.	Six cameras record the movement of the participants’ lower limbs during gait, and the force plates are positioned in the middle of the walkway.	Spatiotemporal parameters, including cadence, step time, stride time, single support, double support, walking speed, step length, stride length, step width, and foot angle.	Symmetry indices may artificially inflate or deflate asymmetry levels due to parameter type or magnitude.
Hatamzadeh et al. [5]	Reference: a 10 m long OptoGait system and a single Microsoft Azure Kinect camera	(spatial resolution: 1.041 cm, temporal resolution: 1000 Hz) 30 Hz.	The camera is placed at a 1 m distance from the end of the OptoGait walkway at a 80 cm height.	Step time, step length, stride time, stride length, and gait speed.	Limitations include validation solely on healthy individuals, limited walking distance (6 m), and unassessed OptoGait biases in phase percentage calculations.
Duncan et al. [24]	Two USB cameras (ZEALINNO 1080P Webcam, Shenzhen MLK Technology Company Limited, Shenzhen, China), one Raspberry Pi V2.1 camera module, and one smartphone.		The physical dimensions of the CBMT platform equal to 13 cm × 19 cm × 36 cm (without the tripod and marker). The gap between the left and right cameras equals 273.5 mm to detect the depth more accurately.	The left and right cameras are used for gait speed tests. The center camera is used for standing balance, 5TSS, and TUG tests.	Limitations include validation only on a small sample size of healthy volunteers and older adults, and there is a reliance on the erratic walking trajectory for balance assessment.
Kanko et al. [25]	Seven Qualisys 3+ cameras (Qualisys AB, Gothenburg, Sweden), which were used to record marker trajectories, and eight Qualisys Miqus cameras	Both systems were recorded at 85 Hz.	The seven Qualisys systems recorded marker trajectories and the eight Qualisys Miqus cameras, which recorded 2D videos, were positioned around an instrumented treadmill.	Step length, stride length, stride width, step time, cycle time, swing time, stance time, double limb support time, and trial-average gait speed.	Limitations include sample bias toward healthy, young individuals and the unknown sensitivity of markerless system to health status and environmental factors.
Keller et al. [26]	Eight Sony RX0II cameras (Sony Corporation, Minato, Japan).	60 frames		Gait speed/step length/stride length/stride width/step time/cycle time/swing time/stance time/double-limb support time	Limitations include restricted clothing conditions and unexplored impact on joint moments, warranting further investigation in clinical populations.
Guillermo et al. [27]	Kinect v2.	30 Hz.	Kinect v2 is placed at 1 m height to make the recording. The person is placed between 1.5 and 4.5 m.	Ankle angle	The method’s significant limitation is its non-real time processing, taking approximately half an hour for one minute of Kinect data, which may hinder timely results acquisition in certain scenarios.
Aung et al. [28]	gold standard measurement: FDM system (Zebris, Germany) and a video camera (Sanyo Xacti VPC-GH1, Vietnam).	100 Hz 60 Hz.	Placed perpendicular to the participants.	Step length, step time, stride length, gait velocity, and cadence.	The study’s limitations include parallax and perspective errors, and a lack of intertester reliability.
Guess et al. [29]	A 12-camera Vicon optical motion capture system and a single Azure Kinect.	100 Hz 30 Hz.	The Azure Kinect DK was placed 2.7 m from the center of the Vicon capture space and 1.0 m above the floor.	Stride length/stride time/step length/step width.	The 30 Hz sample rate limits Azure Kinect’s accuracy at faster speeds and for stance/swing time measurements.
Horsak et al. [30]	A 16-camera motion capture system (Nexus, 2.14, Vicon, Oxford, UK) and two iOS smartphones (iPhone 11 and 12 Pro).	120 Hz 720 × 1280 pixels and 60 Hz.	The cameras were positioned approximately 35 degrees off from the center of the walk way with the lens of each camera at a height of approximately 1.5 m.	Pelvis, hip, knee, and ankle joints.	The study’s limitations include using healthy participants to mimic pathological gait, potential anatomical differences affecting pose estimation, and possible minor variations from cross-correlation data alignment.

**Table 5:** Summary of the participant numbers in various research papers.

Paper Title	Number of Participants	Participant Characteristics
[26]	29 healthy subjects (18 males and 11 females)	Age of 21.5 ± 1.3 (SD) years, mean height of 1.71 ± 0.08 (SD) m, mean mass of 65.03 ± 10.64 (SD) kg, and mean body mass index (BMI) of 22.12 ± 2.44 (SD) kgm^−2^) after excluding subjects wearing inconsistent footwear in both clothing conditions.
[56]	26 initially, but data from 24 were evaluated.	The study included 26 participants: 15 with movement disorders (14 with Parkinson’s Disease and 1 with hereditary spastic spinal paralysis) and 11 without any disorders.
[37]	20 healthy participants (12 males and 8 females)	Twenty healthy participants were included after excluding those with current musculoskeletal issues, prior lower extremity surgery or trauma, or medical conditions impacting movement.
[51]	10 healthy participants	Aged between 18 and 84 years (average age 28.6 years, SD 19.6). The average height was 1.71 m (SD 0.06), and the average weight was 66.8 kg (SD 17.8). Participants were excluded if they had any musculoskeletal disorders.
[57]	32 healthy participants (22 males and 10 females)	They walked along a 6.5 m indoor walkway while recording video and motion capture data. Data from one participant was excluded due to it belonging to a different subset.
[58]	15 healthy participants (7 males [1.82 ± 0.11 m, 85.7 ± 11.1 kg], 8 females [1.65 ± 0.08 m, 63.2 ± 6.0 kg])	In a single session, participants performed ten walking trials, ten running trials, and ten counter-movement jumps in random order, wearing a full-body marker set. Two motion capture systems recorded the movements simultaneously.
[59]	11 subjects (8 males and 3 females)	Age = 24.2 ± 3.8 years, height = 170.7 ± 6.4 cm, and weight = 71.7 ± 16.1 kg) without any presence or history of neurological disorders.
